# Plasma glutamine and glutamic acid are potential biomarkers for predicting diabetic retinopathy

**DOI:** 10.1007/s11306-018-1383-3

**Published:** 2018-06-21

**Authors:** Sang Youl Rhee, Eun Sung Jung, Hye Min Park, Su Jin Jeong, Kiyoung Kim, Suk Chon, Seung-Young Yu, Jeong-Taek Woo, Choong Hwan Lee

**Affiliations:** 1 Department of Endocrinology and Metabolism, Kyung Hee University School of Medicine, 23 Kyungheedae-ro, Dongdaemun-gu, Seoul, 02447 Republic of Korea; 2 Department of Systems Biotechnology, Konkuk University, 120 Neungdong-ro, Gwangjin-gu, Seoul, 05029 Republic of Korea; 3 Statistics Support Department, Kyung Hee University Medical Center Medical Science Research Institute, Seoul, Republic of Korea; 4 Department of Ophthalmology, Kyung Hee University School of Medicine, Seoul, Republic of Korea

**Keywords:** Diabetes mellitus, type 2, Diabetes complications, Diabetic retinopathy, Metabolomics, Glutamine, Glutamic acid

## Abstract

**Introduction:**

Diabetic patients with a long disease duration usually accompanied complication such as diabetic retinopathy, but in some patients had no complication.

**Objectives:**

We analyzed differences in plasma metabolites according to the presence or absence of diabetic retinopathy (DR) in type 2 diabetic (T2D) patients with disease duration ≥ 15 years.

**Methods:**

A cohort of 183 T2D patients was established. Their biospecimens and clinical information were collected in accordance with the guidelines of the National Biobank of Korea, and the Korean Diabetes Association. DR phenotypes of the subjects were verified by ophthalmologic specialists. Plasma metabolites were analyzed using gas chromatography time-of-flight mass spectrometry and ultra-performance liquid chromatography–quadrupole time-of-flight mass spectrometry. And these results were analyzed using multivariate statistics.

**Results:**

For metabolomic study, propensity score matched case and control subjects were chosen. Mean age of the subjects was 66.4 years and mean T2D duration was 22.2 years. Metabolomic identification revealed various carbohydrates, amino acids, and organic compounds that distinguished between age- and sex-matched non-diabetic controls and T2D subjects. Among these, glutamine and glutamic acid were suggested as the most distinctive metabolites for the presence of DR. Receiver operating characteristics curves showed an excellent diagnostic value of combined (AUC = 0.739) and the ratio (AUC = 0.742) of glutamine and glutamic acid for DR. And these results were consistent in validation analyses.

**Conclusion:**

Our results imply that plasma glutamine, glutamic acid, and their ratio may be valuable as novel biomarkers for anticipating DR in T2D subjects.

**Electronic supplementary material:**

The online version of this article (10.1007/s11306-018-1383-3) contains supplementary material, which is available to authorized users.

## Introduction

Diabetic retinopathy (DR) is a chronic complication that is directly referenced in the diagnosis of diabetes mellitus, and is more specific to hyperglycemia than other chronic, diabetes-related complications (Cheung et al. 2010; Song and Wong 2014). If DR is detected early on, proper management can prevent the progression and deterioration of retinopathy; but if the condition is not properly managed, it can lead to severe vision loss or blindness (Cheung et al. 2010; Song and Wong 2014). Currently, DR is regarded as the leading cause of blindness in adults (Cheung et al. 2010; Kempen et al. 2004).

Despite this clinical significance, however, DR is not well screened compared with other complications. According to one study, the screening rate of DR in Korea is 36.3%, which is significantly lower than that for other complications (Byun et al. 2013). Other studies have shown similar results (Fathy et al. 2016; Rhee et al. 2005). The reason for the low screening rate for DR is that it requires additional equipment such as a fundus camera, and it is difficult to perform easily compared with other tests, as it requires skilled medical staff (Byun et al. 2013; Lee and Song 2016; Mukamel et al. 1999).

To improve this situation, it is necessary to identify biomarkers that can screen, judge therapeutic effects, and predict the prognosis of DR. However, studies in related fields have not been successful, and few studies have investigated biomarkers for predicting the outcomes of DR, despite the great clinical importance of this endeavor.

We have constructed and operated a geriatric cohort and a biorepository for patients with type 2 diabetes of over 15 years of disease duration. This study was conducted to identify biomarkers for predicting DR in patients in this cohort undergoing detailed retinopathy phenotyping. We focused in particular on subjects with a long disease duration but no complications, and identified biomarkers that play a protective role in DR and are likely to be associated with its prognosis.

## Methods

### Subjects and study design

This study was conducted as part of the National Biobank project, using the baseline characteristics of prospective cohort study registrants collected from September 2014 to July 2015. The subjects of this cohort were type 2 diabetes patients with a disease duration of 15 years or more.

Clinical information on the subjects was registered based on the standardized methods for multi-center clinical data registration approved by the Korean Diabetes Association, and biospecimens were collected in accordance with the guidelines of the National Biobank of Korea (National Biobank of Korea 2017).

### Ethics statement

This study was approved by the institutional review board of Kyung Hee University Hospital (IRB No. KMC IRB 1428-04). Written informed consent was obtained from all participants. Information on this study was provided by a clinical research information service (CRIS, No. KCT0001269), which is a Korean national service connected with the International Clinical Trials Registry Platform of the World Health Organization (https://cris.nih.go.kr).

### Diabetic retinopathy phenotyping

The DR status of each participant was assessed through color fundus photography (FF 540 Plus; Carl Zeiss Meditech, Jena, Germany) and optical coherence tomography (HD-OCT; Carl Zeiss Meditech, Dublin, CA, USA). In accordance with Early Treatment Diabetic Retinopathy Study (ETDRS) criteria, DR was graded into three categories: no DR, non-proliferative diabetic retinopathy (NPDR), or proliferative diabetic retinopathy (PDR) (Wilkinson et al. 2003; Wu et al. 2013). Two or more ophthalmologists classified the DR status based on the results of the exams. If there was discordance between the evaluators, they reviewed the images and agreed on the final interpretation.

### Statistical analyses for clinical data

In this study, we compared the clinical characteristics of patients with and without DR, focusing on identifying the characteristics of subjects who did not have retinopathy despite a long history of type 2 diabetes. Validation and statistical analyses of the clinical data were performed independently by a statistician. Means, proportions, and distributions of characteristics were compared between patients with or without DR. After initial analysis, case and control sets were selected by propensity score matching (PSM) with similar clinical characteristics aside from DR, and corresponding samples were used for the metabolomic study. SAS software (ver. 9.3; SAS Institute Inc., Cary, NC, USA) was used for all statistical analyses.

### Sample preparation for metabolomic study

Age-matched non-diabetic controls were used for the metabolomic study. The subject’s specimen for metabolomic study was collected by a skilled sampler. And the blood was immediately centrifuged at 4 °C, 1600×*g* for 15 min in an EDTA tube. Then, each 300 µL aliquot was stored at − 70 °C deep freezer. Metabolites were extracted from 200 µL of plasma. A solution of 600 µL of methanol and 10 µL of internal standard solution (2-chlorophenylalanine, 1 mg/mL in water) was added to the serum and then homogenized using a sonicator for 5 min. After homogenization, the suspension was held at − 20 °C for 60 min, and then centrifuged at 12,000 rpm and 4 °C for 10 min. The supernatant was filtered through a 0.2-µm polytetrafluoroethylene (PTFE) filter and dried using a speed vacuum concentrator (Modulspin 31; Biotron, Korea). Dried extracts were re-dissolved in 250 µL of methanol for ultra-performance liquid chromatography–quadrupole/time-of-flight mass spectrometry (UPLC–Q–TOF–MS) analysis, and 100 µL of the samples were dried under a vacuum for gas chromatography (GC)–TOF–MS analysis.

### GC–TOF–MS analysis

For GC–TOF–MS analysis, dried samples were oximated with 50 µL of methoxyamine hydrochloride (20 mg/mL in pyridine) for 90 min at 30 °C, and silylated with 50 µL of *N*-methyl-*N*-(trimethylsilyl) trifluoroacetamide (MSTFA) for 30 min at 37 °C. GC–TOF–MS analysis was performed using an Agilent 7890 gas chromatography system (Agilent Technologies, Palo Alto, CA, USA) coupled with an Agilent 7693 auto-sampler (Agilent Technologies) and equipped with a Pegasus® HT TOF MS (LECO Corp., St. Joseph, MI, USA) system. An Rtx-5MS column (i.d., 30 m × 0.25 mm, 0.25 µm particle size; Restek Corp., Bellefonte, PA, USA) was used with a constant flow of 1.5 mL/min of helium as the carrier gas. Samples (1-µL aliquots) were injected into the GC with splitless mode. The oven temperature was maintained at 75 °C for 2 min, then incrementally raised 15 °C/min to 300 °C, and finally held for 3 min. The temperatures of the front inlet and transfer line were 250 and 240 °C, respectively. The electron ionization was carried out at − 70 eV and full scanning over the range of 50–1000 *m*/*z* was used for mass data collection.

### UPLC–Q–TOF–MS analysis

UPLC was performed on a Waters ACQUITY UPLC™ system (Waters Corp., Milford, MA, USA) equipped with a binary solvent delivery system, a UV detector, and an auto-sampler. Chromatographic separation was performed on a Waters ACQUITY BEH C18 column (i.d., 100 mm × 2.1 mm, 1.7 µm particle size; Waters Corp.) and the injection volume was 5 µL. The column temperature was set at 37 °C and the flow rate was 0.3 mL/min. The mobile phase consisted of 0.1% v/v formic acid in water (A) and 0.1% v/v formic acid in acetonitrile (B). The initial condition was 5% B for 1 min and linearly increased to 100% B over 9 min. Total run time was 14 min including re-equilibration of the column to the initial conditions. For MS experiments, the Waters Q–TOF Premier (Micromass MS Technologies, Manchester, UK) was operated in negative ion mode with an *m*/*z* range of 100–1000. The source temperature was set at 100 °C, the collision energy was set at 10 eV, the collision gas flow was 0.3 mL/min, and the desolvation gas was set to 650 L/h at a temperature of 300 °C. The capillary voltage and sample cone voltage were set at 2.5 kV and 50 V, respectively. The V mode was used for the mass spectrometer and data were collected in the centroid mode with a scan accumulation of 0.2 s. Leucine encephalin was used as reference lock mass (*m*/*z* 554.2615) by independent LockSpray interference.

### Data processing and multivariate statistical analysis for metabolomic study

The GC–TOF–MS data were acquired and preprocessed using the LECO Chroma TOF™ software (version 4.44, LECO Corp.) and converted into the NetCDF format (*.cdf) using the LECO Chroma TOF™ software. The raw data from UPLC–Q–TOF–MS analysis were acquired by MassLynx software (version 4.1, Waters Corp.). Raw data files were converted into the NetCDF format (*.cdf) using the MassLynx DataBridge software (version 4.1, Waters Corp.). After conversion, peak detection, retention time correction, and alignment were processed using the Metalign software package (http://www.metalign.nl). The resulting data were exported to a Microsoft Excel file. Multivariate statistical analysis was conducted using SIMCA-P+ (version 12.0; Umetrics, Umeå, Sweden). The data sets were auto-scaled (unit variance scaling) and mean-centered in a column-wise fashion. Principal component analysis (PCA) and orthogonal partial least squares–discriminant analysis (OPLS–DA) were performed to compare each data set. The variables were selected based on variable importance to projection (VIP) values of the OPLS–DA. Significant differences were determined by analysis of variance (ANOVA), Student’s t-test, and Duncan’s multiple range tests using PASW Statistics 18 software (SPSS Inc., Chicago, IL, USA). The box and whisker plots were rendered using the relative peak area of unique masses of metabolites by STATISTICA 7 software (StatSoft Inc., Tulsa, OK, USA). Receiver operating characteristic (ROC) curves and logistic regression analysis were generated by using Medcalc software (version 14.8.1; Medcalc Software, Mariakerke, Belgium).

### Validation by amino acid quantification

Quantification was performed using an amino acid analyzer (Biochrom 30+; Biochrom Ltd., Cambridge, UK) to confirm the metabolomic study results. Pairs of case and control sets (n = 48) were selected again through PSM.

## Results

### Study progression and characteristics after PSM

Of the 220 subjects recruited to the study, clinical data and samples were collected from 198 subjects who provided consent. After the withdrawal of 15 subjects, a total of 183 completed ophthalmologic exams (Figure S1). The mean age of the participants was 66.8 years, the median duration of diabetes mellitus was 22.6 years, and 49.7% were male. Among a total of 183 participants who underwent ophthalmologic assessment, 124 (67.8%) were diagnosed with DR; 72 (39.3%) had NPDR and 52 (28.4%) had PDR. Statistically significant differences were found for several factors. PSM was performed based on these data, and 32 pairs of cases and controls with no significant differences in clinical characteristics except for the presence or absence of DR were selected (Table S1). These pairs were used for the metabolomic study.

### Plasma metabolite differences between subjects grouped by DR status

Significantly discriminated non-targeted metabolites among non-diabetic controls and subjects with no DR, NPDR, or PDR were investigated to discover biomarkers of diabetes and DR. Plasma samples were analyzed using GC–TOF–MS and UPLC–Q–TOF–MS; 39,154 and 6185 mass spectral variables, respectively, were used for further multivariate analysis.

**Fig. 1:**
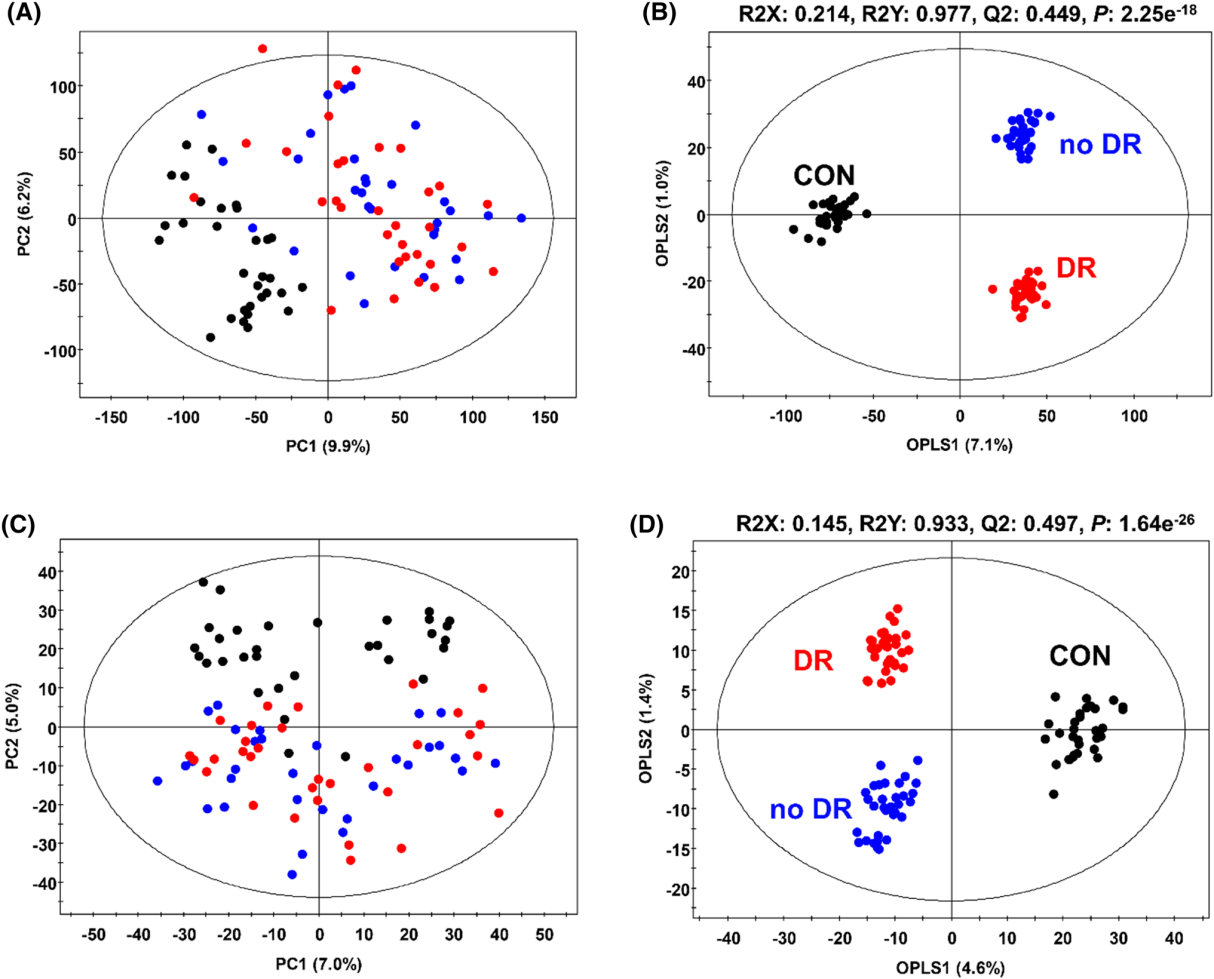
Principal component analysis (PCA) (**a, c**) and orthogonal partial least squares discriminant analysis (OPLS–DA) (**b, d**) score plots for plasma of non-diabetic control, no DR, and DR subjects analyzed by GC–TOF–MS (**a, b**), and UPLC–Q–TOF–MS (**c, d**)

In the PCA with score plots derived from GC–TOF–MS data sets, the non-diabetic control group was clearly clustered from other groups along with PC1 (9.9%), while the groups of patients with diabetes and DR were not clearly separated from each other (Fig. 1a). However, when we applied an orthogonal partial least squares discriminant analysis (OPLS–DA) model with supervised methods, all three groups were clearly separated from each other with model values of R^2^X_(cum)_ = 0.214, R^2^Y_(cum)_ = 0.977, and Q^2^_(cum)_ = 0.449, which indicated the fitness and prediction accuracy of the model (Fig. 1b). The quality of the model was evaluated by cross-validation analysis (*p*-value = 2.25e^−18^).

Similar distribution patterns were observed in the PCA and OPLS–DA of the UPLC–Q–TOF–MS data sets. In the PCA, the non-diabetic control group was discriminated from the other groups by PC2 (5.0%). However, the non-DR and DR subgroups were undifferentiated (Fig. 1c). In the OPLS–DA score plots, the non-diabetic control group and subjects with and without DR were discriminated from each other with model values of R^2^X_(cum)_ = 0.145, R^2^Y_(cum)_ = 0.933, and Q^2^_(cum)_ = 0.497, and the *p*-value = 1.64e^−26^ was obtained from cross-validation analysis (Fig. 1d).

To select the metabolites responsible for these separations, VIP values > 0.7 of OPLS–DAs were used. The VIP value is an important parameter for detecting potential biomarker candidates that reflects the correlation of the metabolites with different biological states. For evaluating statistical significance, *p* < 0.05 derived from t-test was applied.

Selected metabolites were identified by comparing MS fragment patterns with commercial standard compounds and various databases, including the National Institutes of Standards and Technology (NIST) library, the Human Metabolome Database (HMDB, http://www.hmdb.ca/), and Wiley 8. The detailed information of these metabolites is presented in Tables S2 and S3. A total of 31 metabolites, including 7 amino acids, 6 organic compounds, 7 carbohydrates, and 11 lysophosphatidylcholines (lysoPCs), were identified as metabolites that differed significantly among the experimental groups. The relative metabolite levels were converted into fold changes and are also presented in Tables S2 and S3.

### Combination of multi-metabolite biomarkers for the diagnosis of diabetes and diabetic retinopathy

Box and whisker plots and ROC curves were constructed for the selected 31 metabolites using the relative metabolite contents of the experimental groups (Figs. S2–S5). Twenty-eight of the metabolites showed good discriminatory power for non-diabetic versus diabetic subjects, with an area under the curve (AUC) > 0.7, except for urea (**9**) and two lysoPCs of C14:0 (**21**) and C20:3 (**29**) (Tables S2, S3 and Figs. S2–S5).

Among the assigned metabolites, several amino acids and carbohydrates showed dramatic increases and decreases in diabetic subjects compared with their levels in non-diabetic controls. Asparagine (2.30-fold, **6**), glutamine (2.83-fold, **7**), fructose (2.02-fold, **15**), and *myo*-inositol (2.02-fold, **20**) were markedly increased in diabetic subjects, while aspartic acid (0.46-fold, **2**), glutamic acid (0.25-fold, **5**), and 1,5-anhydroglucitol (0.25-fold, **14**) were markedly decreased.

**Fig. 2:**
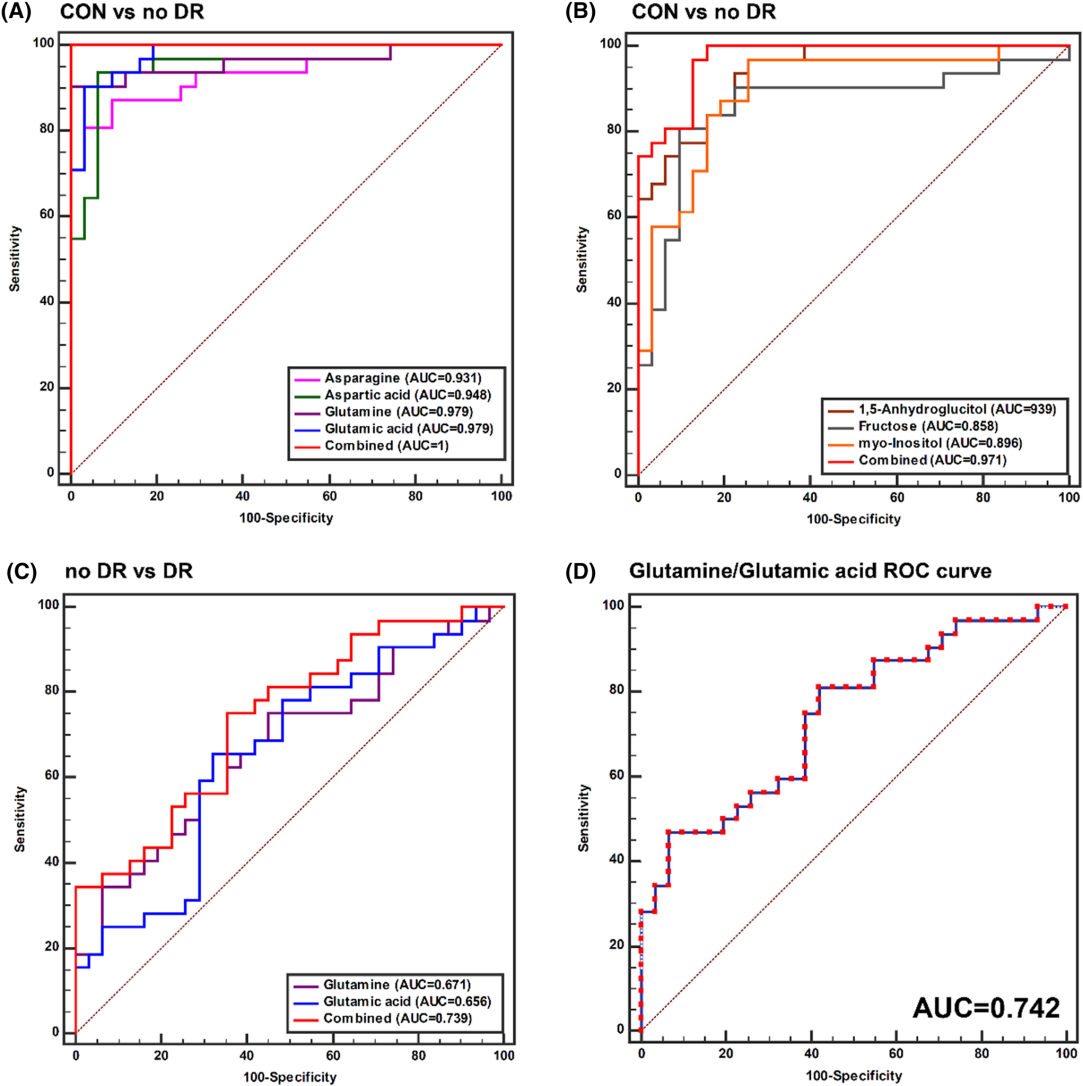
Combined ROC curves of potential metabolite biomarkers distinguishing non-diabetic versus diabetic subjects and no DR versus DR subjects. **a** Four amino acids which potential metabolite biomarkers that shows most high fold changes between non-diabetic and diabetic subjects were combined. **b** Three carbohydrates potential metabolite biomarkers that shows most high fold changes between non-diabetes and diabetes patients were combined. **c** Two amino acids which potential metabolite biomarkers that shows significance of changes between no DR and DR subjects were combined. **d** ROC curve of glutamine to glutamic acid ratio. The ROC curves of each metabolites and combined ROC curves were overlain on single plots. The AUC values of each metabolites are shown in inside of ROC curve

The combination of four of these amino acids, asparagine (**6**), aspartic acid (**2**), glutamine (**7**), and glutamic acid (**5**), highly improved the specificity in distinguishing diabetic subjects from non-diabetic controls, with a combined AUC value of 1.00 (Fig. 2a). The combination of three carbohydrates, namely, 1,5-anhydroglucitol (**14**), fructose (**15**), and *myo*-inositol (**20**), yielding a combined AUC value of 0.971, also improved the power in discriminating between diabetic subjects and non-diabetic controls (Fig. 2b). Further, glutamic acid (**5**) and glutamine (**7**) were significantly decreased (0.72-fold) and increased (1.19-fold) in DR subjects, respectively (Table S2). However, these metabolites showed poor discriminatory power for discriminating between subjects with and without DR, with an AUC of 0.656.

The combination of those DR subjects’ specific metabolites improved the power to discriminate DR subjects from diabetic patients, with a combined AUC value of 0.739 (Fig. 2c). To maximize the differences in metabolite levels between patients with and without DR, the ratio of the levels of glutamine to glutamic acid was calculated (Table 1). This ratio was significantly different among the experimental groups: 1.31 for non-diabetic controls, 8.79 for diabetic patients without DR, and 13.24 for patients with DR. ROC curve analysis was performed using the glutamine/glutamic acid ratios of subjects with and without DR, for an AUC of 0.742 (Fig. 2d). According to these results, the glutamine/glutamic acid ratio was the best biomarker for distinguishing patients with DR among diabetic subjects.

**Table 1:** Mean concentrations of potential metabolite biomarkers distinguishing non-diabetic control, no DR, and DR subjects as quantified by GC–TOF–MS analysis

No.	Metabolite	Mean concentration (ng/80 µL serum)		
		CON	No DR	DR
1	Asparagine	8.06 ± 0.38	9.56 ± 1.37*	9.80 ± 1.08
2	Aspartic acid	10.01 ± 0.33	9.44 ± 0.19*	9.40 ± 0.14
3	Glutamine	52.62 ± 20.91	135.36 ± 35.74*	160.05 ± 40.49^#^
4	Glutamic acid	50.17 ± 21.09	16.62 ± 5.97*	13.50 ± 4.81^#^
5	Glutamine/glutamic acid	1.31	8.79*	13.24^#^

*p-value < 0.05 by t-test between CON and No DR groups

^#^*p*-value < 0.05 by t-test between No DR and DR groups

### Amino acid quantification for validation

Amino acid quantification was performed on 48 pairs of non-DR and DR subjects. As a result, the plasma glutamine concentration in non-DR subjects was 430.17 ± 115.91 and 489.19 ± 90.59 nmol/mL in DR subjects (*p* = 0.002, by t-test). The concentration of glutamic acid in non-DR subjects was 165.86 ± 62.55 and 150.16 ± 63.99 nmol/mL in DR subjects (*p* = 0.242). The glutamine/glutamic acid ratio was 3.00 ± 1.46 in non-DR subjects and 3.77 ± 1.51 in DR subjects (*p* = 0.013), consistent with the results from the metabolomic study.

## Discussion

Metabolomics is a powerful approach for studying pathophysiological processes, and has been used to identify complex endogenous metabolic phenotypes in various diseases such as diabetes mellitus. Diseases can be characterized by metabolic alterations in key regulatory pathways. Diabetes-related complications such as retinopathy are intensified due to dysfunctions in multiple metabolic pathways (Filla and Edwards 2016; Sas et al. 2015).

**Fig. 3:**
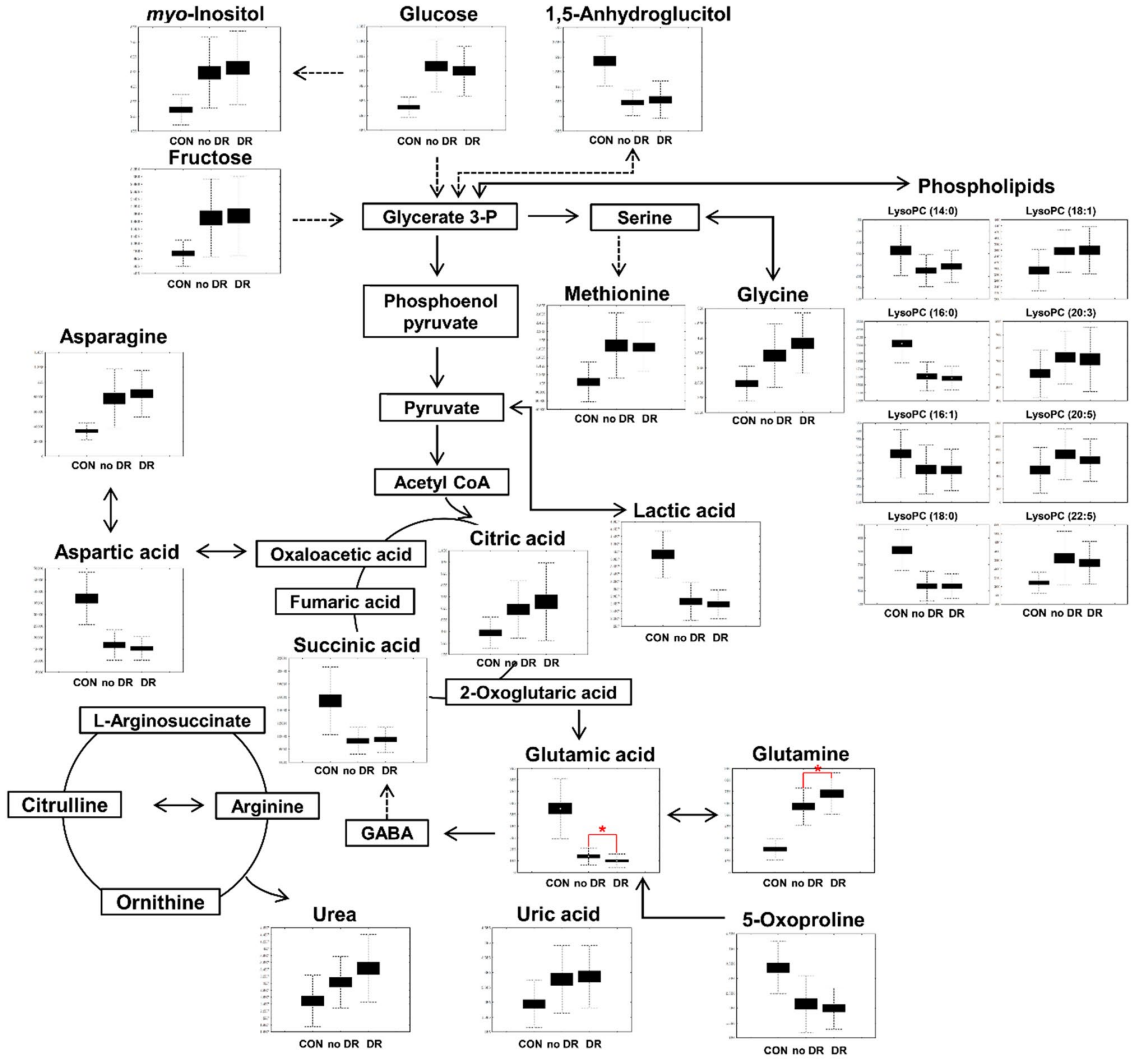
A schematic diagram of a proposed metabolic pathway using metabolites shows significantly different levels among experimental groups including non-diabetic control, no DR, and DR subjects

From the plasma metabolomic analysis of non-diabetic controls and diabetic patients with and without DR, various metabolites were selected as candidate biomarkers by multivariate analysis, and we generated a metabolic pathway to show the relationships among these metabolites (Fig. 3). These metabolites belong to pathways of amino acid, energy, carbohydrate, and lipid metabolism, which are known to be closely related to insulin resistance and secretion (Floegel et al. 2013; Greenfield et al. 2009; Koves et al. 2008; Montonen et al. 2007). The relative levels of these metabolites were dramatically different between non-diabetic controls and the other experimental groups.

The large differences may be due to the duration of diabetes; in this study, patients both with and without DR had suffered from type 2 diabetes for longer than 20 years, and metabolic changes persistently occurred with this long-standing disease. Among these metabolic changes, four amino acids, asparagine (**6**), aspartic acid (**2**), glutamine (**7**), and glutamic acid (**5**), and three carbohydrates, 1,5-anhydroglucitol (**14**), fructose (**15**), and *myo*-inositol (**20**), differed significantly between diabetic patients and non-diabetic subjects (Table S2 and Fig. 3).

ROC curves are the most common tool for evaluating prediction power, and AUC is used for measuring prediction. These kinds of prediction metrics can provide insights into the use of metabolite biomarkers in disease prediction (Klein and Shearer 2016). The combined AUC of the four amino acids and three carbohydrates was 1.00 and 0.971, respectively (Fig. 2a, b). From these prediction models from multivariate analysis and ROC curve construction, the combination of these multiple metabolite biomarkers could provide improved specificity compared with single-metabolite biomarkers for distinguishing diabetic patients from non-diabetic controls.

The key finding of the current study was the identification of a blood-derived biomarker that distinguishes DR cases among long-standing type 2 diabetes patients. Only a few studies have considered investigating metabolic markers of DR. However, most of these studies were limited to using vitreous humor (Filla et al. 2014; Klein and Shearer 2016; Paris et al. 2016). Invasive procedures are required for obtaining vitreous humor, so it cannot be used in a general outpatient setting. For these reasons, it is necessary to identify biomarkers of diagnostic value in easily accessible samples such as blood.

Based on our results, plasma glutamine and glutamic acid were selected as biomarker candidates for distinguishing DR cases. The ROC curve analysis revealed that the combination of these two metabolites improved specificity, but a more improved result was obtained by using the value of the glutamine/glutamic acid ratio to discriminate between patients with and without DR (Fig. 2c, d).

The roles of plasma glutamine and glutamic acid in DR have not yet been elucidated. However, there is some evidence for their involvement in the development of diabetes and diabetes-related complications. Cheng et al. reported that plasma glutamine, glutamate, and the glutamine/glutamate ratio were strongly associated with insulin resistance traits in two different cohorts, the Framingham Heart Study and the Malmö Diet and Cancer Study (Cheng et al. 2012). In particular, this study revealed an association between a high glutamine/glutamate ratio and lower risk of diabetes incidence. However, this pattern was inconsistent between the cohorts (Cheng et al. 2012). In addition, similarly altered patterns of plasma amino acids, including glutamine, asparagine, and aspartic acid, in diabetic patients compared with non-diabetic subjects were also reported in another study (Zhou et al. 2013). Furthermore, glutamine/glutamic acid metabolism connected to various cellular functions such as protein synthesis, muscle growth, ureogenesis in the liver, insulin secretion in pancreatic β-cell, hepatic and renal gluconeogenesis, neurotransmitter synthesis, and glutathione production (Newsholme et al. 2003). Particularly, regulation of insulin secretion from pancreatic β-cell is considered as targets for diabetes therapies (Newsholme et al. 2005). In β-cell, glutamine is carried by blood and accumulated on plasma membrane, then further converted to glutamic acid (Jenstad and Chaudhry 2013). Through the interconnected coupling mechanisms between various molecules and enzymes such as glucose, glutamine, glutamic acid, leucine, GABA, and glutamate dehydrogenase, insulin secretion is regulated. The glutamic acid concentration is also one of the most important indicators of diabetic retinas. Many other studies have suggested that diabetes is accompanied by an accumulation of glutamate in the retina, which causes neurotoxicity and the development of DR (Kowluru et al. 2001; Li and Puro 2002; Lieth et al. 2000). Although, a direct relationship between retinal glutamic acid and plasma glutamic acid has not been well studied, we could carefully suggested that plasma glutamine/glutamic acid level may have close relation with insulin secretion and retina accumulation.

The results of our study have some notable differences from previous studies. This study was conducted by distinguishing between diabetic patients with and without DR, even though the mean disease duration was more than 20 years. This study design was based on the assumption that people who have not had complications even after a long period of type 2 diabetes possess genetic or environmental protective characteristics. Therefore, the present study may have important implications for understanding the pathophysiology of the development and progression of diabetic complications and for determining and predicting the long-term prognosis of DR.

The study also involved the meticulous collection of data from subjects based on consensus-based clinical registration forms and national biospecimen collection guidelines. The collected data were used as optimal conditions for significant metabolite screening by controlling the clinical variables as much as possible through PSM. Therefore, even though a small number of subjects were used for the study, markers with high diagnostic value could be screened. To our knowledge, this study is the first to confirm that glutamic acid and glutamine are closely related to DR outcome in humans.

The limitations of this study are that it was a single-center study, so the number of subjects was rather small; and cross-sectional analysis using baseline data made it difficult to identify causal relationships. However, we are recruiting more patients based on this study and continue to follow their clinical courses. If future research findings accumulate, we believe that we can overcome the limitations of the current study design and obtain a more detailed basis for DR and the microvascular complications of type 2 diabetes.

In conclusion, this study suggests that metabolic biomarkers of DR, especially the glutamine/glutamate ratio, could be used as indicators for the long-term prognosis associated with DR in long-standing type 2 diabetes patients.

## Electronic supplementary material

Below is the link to the electronic supplementary material.

Supplementary material 1 (DOCX 1505 KB)

Supplementary material 2 (DOCX 33 KB)
