# Supplementary material for: Plasma glutamine and glutamic acid are potential biomarkers for predicting diabetic retinopathy
Source: Metabolomics. 2018 Jun 21;14(7):89. doi: 10.1007/s11306-018-1383-3 (PMC6013531; doi:10.1007/s11306-018-1383-3)

**Supplement Figures**

**Figure S1.** Study Progression

**
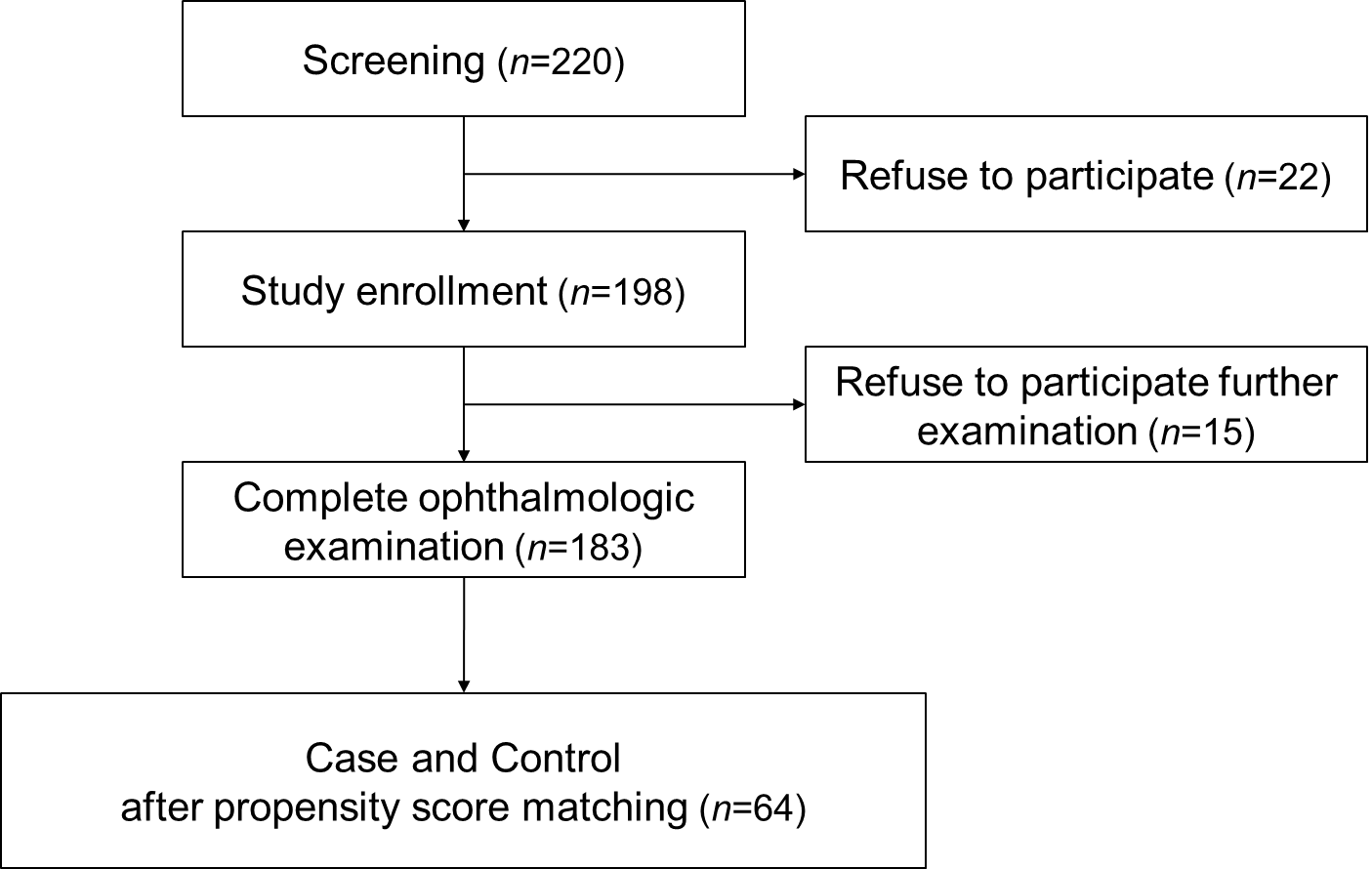
**

**Figure S2.** Box and whisker plots and ROC curves of amino acids in plasma which significantly distinguish non-diabetic control, no DR, and DR subjects. The AUC values of each metabolites are shown in inside of ROC curve.


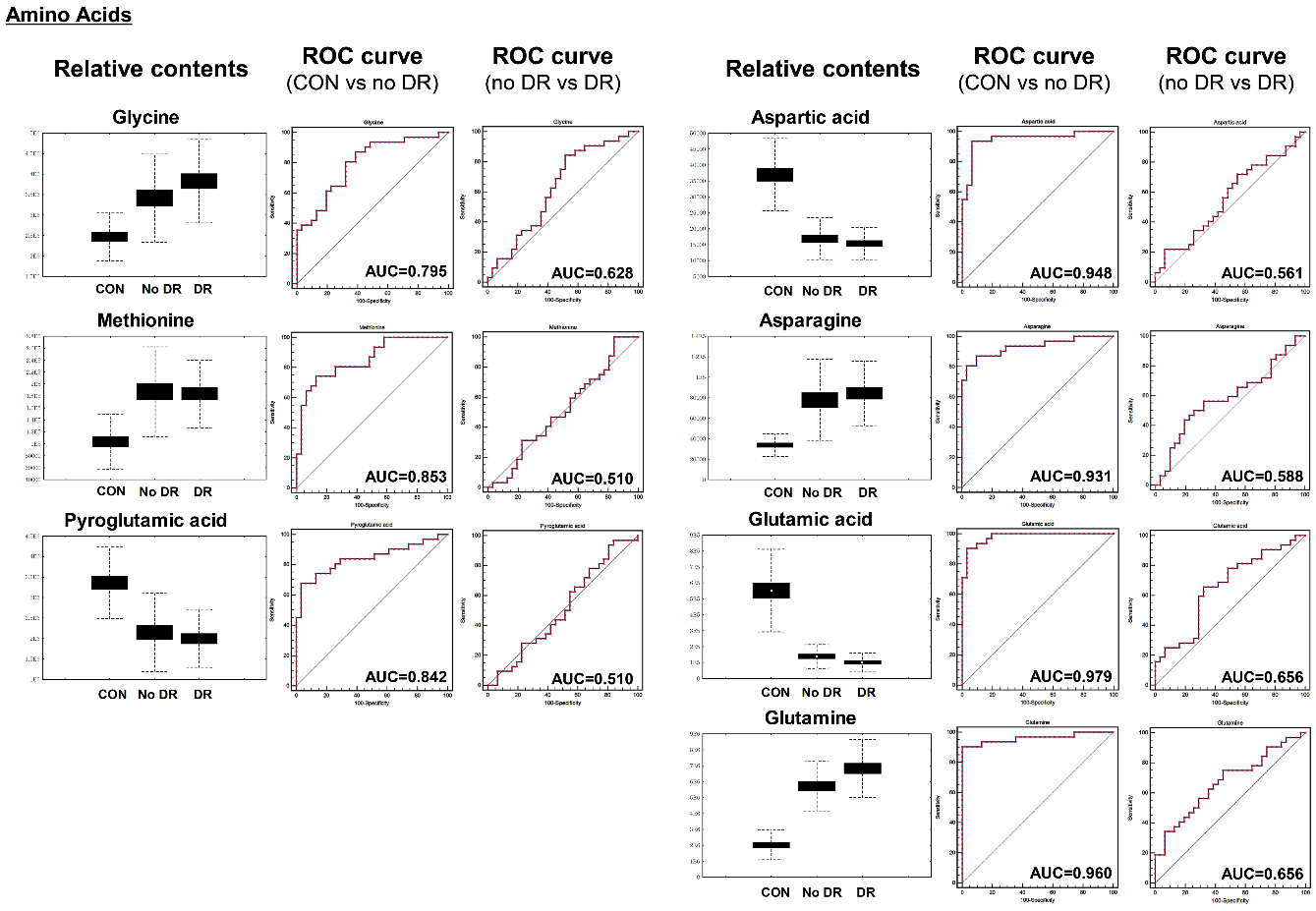


**Figure S3.** Box and whisker plots and ROC curves of organic compounds in plasma which significantly distinguish non-diabetic control, no DR, and DR subjects. The AUC values of each metabolites are shown in inside of ROC curve.


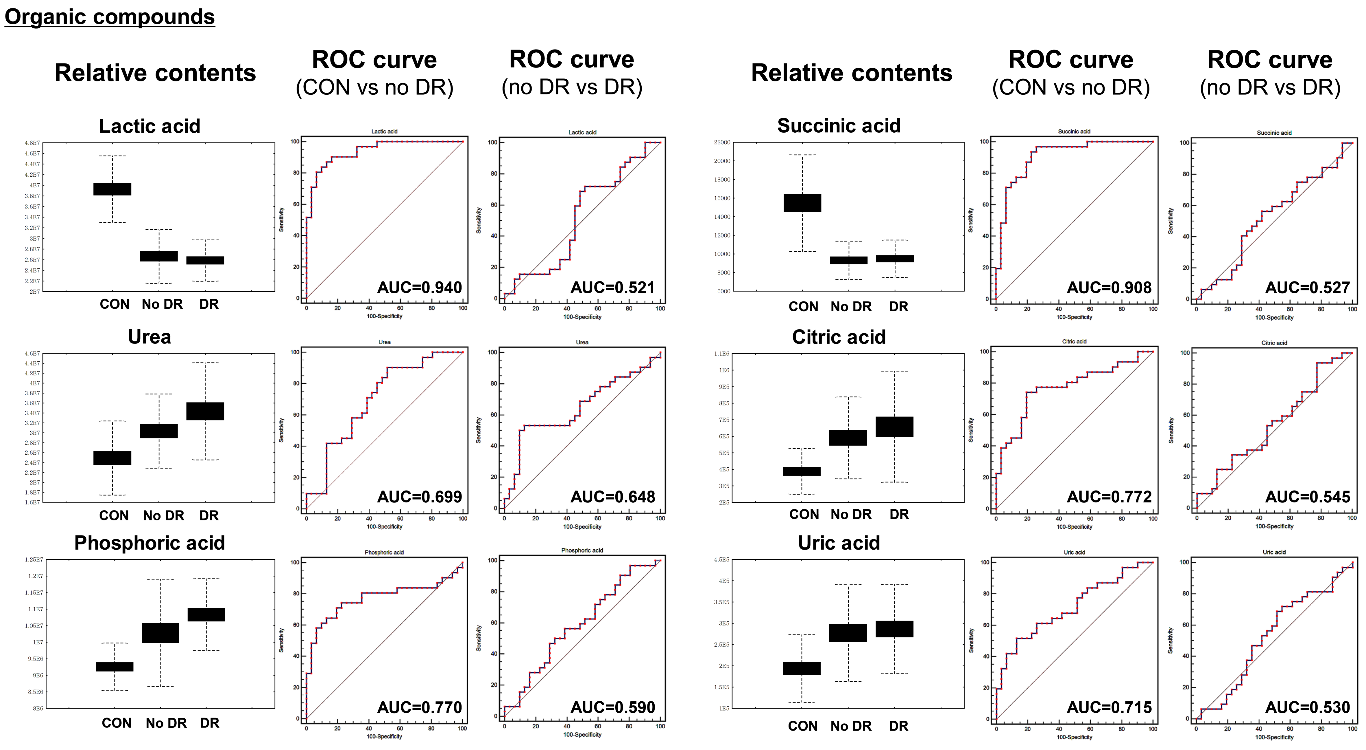


**Figure S4.** Box and whisker plots and ROC curves of carbohydrates in plasma which significantly distinguish non-diabetic control, no DR, and DR subjects. The AUC values of each metabolites are shown in inside of ROC curve.

**
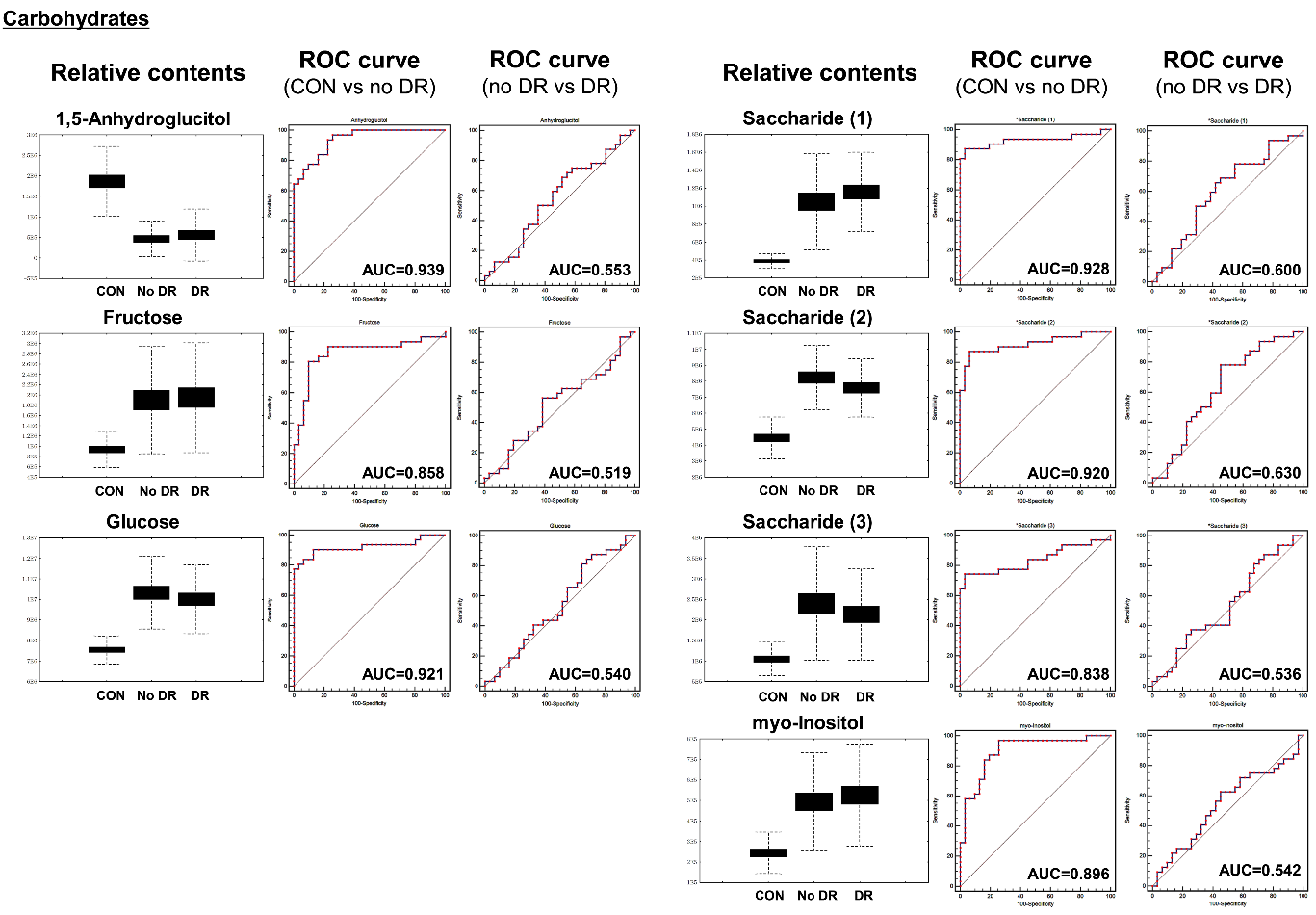
**

**Figure S5.** Box and whisker plots and ROC curves of lysophospholipids in plasma which significantly distinguish non-diabetic control, no DR, and DR subjects. The AUC values of each metabolites are shown in inside of ROC curve.


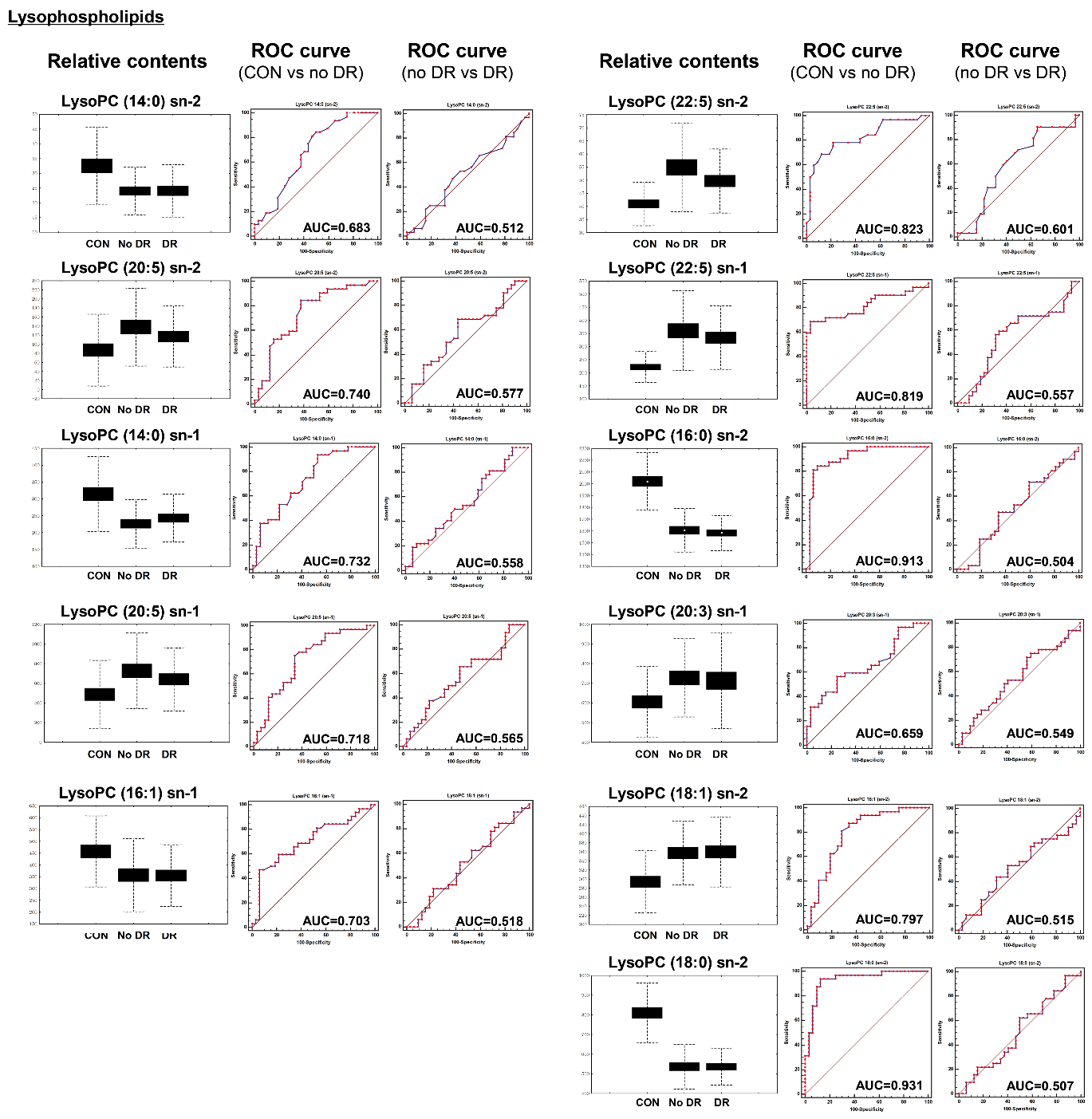

Supplement: Supplementary file 1 — Supplementary material 1 (DOCX 1505 KB) [file 11306_2018_1383_MOESM1_ESM.docx]
