# Supplementary material for: Plasma glutamine and glutamic acid are potential biomarkers for predicting diabetic retinopathy
Source: Metabolomics. 2018 Jun 21;14(7):89. doi: 10.1007/s11306-018-1383-3 (PMC6013531; doi:10.1007/s11306-018-1383-3)
Supplement: Supplementary file 2 — Supplementary material 2 (DOCX 33 KB) [file 11306_2018_1383_MOESM2_ESM.docx]

**Supplement Table**

**Table S1.** Clinical characteristics of the subjects after propensity score matching

| Category | Variables | No DR (n=32) | DR (n=32) | *p* |
| --- | --- | --- | --- | --- |
| Clinical characteristics | Gender (male, %) | 17 (53.1) | 12 (37.5) | 0.383 |
|  | DM duration (yr) | 21.72±6.63 | 22.66±7.77 | 0.624 |
|  | Age (yrs) | 65.25±8.06 | 67.59±8.47 | 0.312 |
|  | Height (cm) | 161.81±8.9 | 162.3±9.14 | 0.850 |
|  | Weight (kg) | 64.1±8.94 | 62.98±12.49 | 0.684 |
|  | BMI (kg/m^2^) | 24.5±3.01 | 23.75±3.32 | 0.375 |
|  | Max weight in lifetime (kg) | 70.65±11.13 | 70.04±11.34 | 0.813 |
|  | Waist circumference (cm) | 88.91±7.74 | 87.74±9.65 | 0.759 |
|  | Hip circumference (cm) | 92.62±5.53 | 92.25±8.03 | 0.953 |
|  | Neck circumference (cm) | 35.89±2.74 | 34.92±3.48 | 0.220 |
|  | Chest circumference (cm) | 92.67±5.39 | 91.33±8.05 | 0.440 |
|  | Thigh circumference (Lt., cm) | 49.36±2.69 | 49.04±3.65 | 0.634 |
|  | Thigh circumference (Rt., cm) | 49.72±3.03 | 49.54±4.01 | 0.771 |
|  | Systolic blood pressure (mmHg) | 127.56±15.09 | 125.66±14.06 | 0.573 |
|  | Diastolic blood pressure (mmHg) | 72.91±10.59 | 68.62±9.67 | 0.073 |
|  | Pulse rate (bpm) | 79.94±15.11 | 81.47±12.94 | 0.682 |
|  | HbA1c (%) | 7.92±1.49 | 7.83±1.19 | 0.778 |
|  | Fasting plasma glucose (mg/dL) | 151.81±57.22 | 143.5±41.24 | 0.524 |
|  | Total cholesterol (mg/dL) | 178.28±34.26 | 161.62±35.84 | 0.052 |
|  | Triglyceride (mg/dL) | 129.47±91.91 | 104.62±52.57 | 0.181 |
|  | LDL cholesterol (mg/dL) | 103.78±27.66 | 95.38±31.56 | 0.235 |
|  | HDL cholesterol (mg/dL) | 55.91±16.86 | 50.19±14.63 | 0.168 |
|  | Spot urine Microalbumin (ug/mg Cr) | 77.83±221.27 | 71.96±175.21 | 0.911 |
|  | BUN (mg/dL) | 16.41±5.78 | 18.69±5.37 | 0.071 |
|  | Creatinine (mg/dL) | 0.8±0.28 | 0.83±0.36 | 0.705 |
|  | Creatinine Clearance (mL/min/1.73m^2^) | 98.04±31.23 | 102.16±40.51 | 0.640 |
|  | AST (IU/L) | 24.34±9.67 | 23.53±6.33 | 0.690 |
|  | ALT (IU/L) | 22.22±16.81 | 22.19±11.87 | 0.994 |
|  | GGT (IU/L) | 30.5±16.27 | 34.19±43.47 | 0.659 |
|  | ALP (IU/L) | 74.19±23.67 | 76.25±17.06 | 0.681 |
| History of Macrovascular complication | Hypertension (n, %) | 23 (71.9) | 28 (87.5) | 0.228 |
|  | Dyslipidemia (n, %) | 22 (68.8) | 21 (65.6) | 1.000 |
|  | Myocardial infarction (n, %) | 0 (0.0) | 0 (0.0) | - |
|  | Angina (n, %) | 3 (9.4) | 2 (6.3) | 1.000 |
|  | Heart failure (n, %) | 0 (0.0) | 1 (3.1) | - |
|  | Atrial fibrillation (n, %) | 0 (0.0) | 0 (0.0) | - |
|  | Any Stroke, (n, %) | 5 (15.6) | 4 (12.5) | 1.000 |
|  | Peripheral arterial disease (n, %) | 2 (6.3) | 2 (6.3) | 1.000 |
|  | Coronary artery ballooning (n, %) | 1 (3.1) | 0 (0.0) | - |
|  | CAG with stent insertion (n, %) | 1 (3.1) | 1 (3.1) | 1.000 |
|  | Coronary artery bypass graft (n, %) | 0 (0.0) | 0 (0.0) | - |
|  | Intervention for peripheral arterial disease (n, %) | 0 (0.0) | 0 (0.0) | - |
|  | Amputation (n, %) | 0 (0.0) | 0 (0.0) | - |
| History of microvascular complication | Glaucoma (n, %) | 4 (12.5) | 4 (12.5) | 1.000 |
|  | Cataract (n, %) | 15 (46.9) | 16 (50.0) | 1.000 |
|  | Blindness (n, %) | 0 (0.0) | 1 (3.1) | - |
|  | Microalbuminuria (n, %) | 12 (37.5) | 9 (28.1) | 0.579 |
|  | Overt proteinuria (n, %) | 6 (18.8) | 4 (12.5) | 0.752 |
|  | Chronic Kidney Disease (n, %) | 6 (18.8) | 2 (6.3) | 0.289 |
|  | Peripheral neuropathy (n, %) | 16 (50.0) | 16 (50.0) | 1.000 |
|  | Autonomic neuropathy (n, %) | 7 (21.9) | 11 (34.4) | 0.343 |
|  | Photocoagulation (n, %) | 0 (0.0) | 10 (31.3) | - |
|  | Intra-vitreal injection (n, %) | 0 (0.0) | 1 (3.1) | - |
|  | Ophthalmologic operation (n, %) | 11 (34.4) | 14 (43.8) | 0.606 |
|  | Dialysis (n, %) | 0 (0.0) | 0 (0.0) | - |
| History of Other comorbidity | Any Cancer (n, %) | 6 (18.8) | 2 (6.3) | 0.289 |
|  | Auto-immune disease (n, %) | 1 (3.1) | 1 (3.1) | 1.000 |
|  | Chronic B viral hepatitis (n, %) | 1 (3.1) | 2 (6.3) | 1.000 |
|  | Chronic C viral hepatitis (n, %) | 0 (0.0) | 0 (0.0) | - |
|  | Liver cirrhosis (n, %) | 0 (0.0) | 0 (0.0) | - |
|  | Tuberculosis (n, %) | 2 (6.3) | 0 (0.0) | - |
|  | Hyperthyroidism (n, %) | 1 (3.1) | 1 (3.1) | 1.000 |
|  | Hypothyroidism (n, %) | 6 (18.8) | 3 (9.4) | 0.505 |
|  | Depression (n, %) | 5 (15.6) | 1 (3.1) | 0.134 |
|  | Periodontitis (n, %) | 5 (15.6) | 7 (21.9) | 0.724 |
| Family history | Family history of DM (n, %) | 22 (68.8) | 24 (75.0) | 0.789 |
|  | Family history of hypertension (n, %) | 10 (31.3) | 9 (28.1) | 1.000 |
|  | Family history of dyslipidemia (n, %) | 0 (0.0) | 1 (3.1) | 1.000 |
|  | Family history of obesity (n, %) | 6 (18.8) | 6 (18.8) | 1.000 |
|  | Family history of cardiovascular disease (n, %) | 0 (0.0) | 1 (3.1) | - |
|  | Family history of stroke (n, %) | 7 (21.9) | 3 (9.4) | 0.343 |
|  | Family history of any cancer (n, %) | 10 (31.3) | 7 (21.9) | 0.579 |
|  | Family history of depression (n, %) | 0 (0.0) | 2 (6.3) | - |
|  | Family history of early (or sudden) death (n, %) | 1 (3.1) | 2 (6.3) | 1.000 |
| Lifestyle | Marriage (n, %) | 32 (100.0) | 32 (100.0) | - |
|  | Monthly income (10,000 KRW) | 247.85±289.62 | 301.46±336.16 | 0.858 |
|  | Regular exercise (n/week) | 3.47±2.87 | 2.44±2.6 | 0.104 |
|  | Average sleep hour within 1 year (hour) | 6.69±1.4 | 6.25±2.09 | 0.246 |
|  | Average frequency of regular meal (n/wk) | 2.88±0.34 | 2.97±0.18 | 0.184 |
|  | Skip breakfast (n/wk) | 0.47±1.57 | 0.44±1.72 | 0.942 |
|  | Average Meal time (min) | 16.09±7.48 | 14.38±8.01 | 0.400 |
|  | Eating out (n/wk) | 2.06±2.94 | 1.94±2.31 | 0.858 |
|  | Night time worker (n, %) | 2 (6.3) | 5 (15.6) | 0.450 |
|  | Self-monitoring of blood glucose (n, %) | 18 (56.3) | 18 (56.3) | 1.000 |
|  | Average number of glucose monitoring (n/wk) | 4.56±2.64 | 3.61±2.64 | 0.283 |
|  | Hypoglycemia (n/month) | 0.56±1.29 | 1.17±2.43 | 0.819 |
|  | DM education within 1 year (n, %) | 0 (0.0) | 3 (9.4) | - |
|  | Any hospitalization within 1 year (n, %) | 9 (28.1) | 8 (25.0) | 1.000 |
|  | Any operation within 1 year (n, %) | 5 (15.6) | 4 (12.5) | 1.000 |
|  | Any ED visit within 1 year (n, %) | 3 (9.4) | 5 (15.6) | 0.724 |
|  | Any dietary supplements within 1 year (n, %) | 16 (50.0) | 15 (46.9) | 1.000 |
| Current Medications | Metformin (n, %) | 23 (71.9) | 23 (71.9) | 1.000 |
|  | Sulfonylurea (n, %) | 20 (62.5) | 20 (62.5) | 1.000 |
|  | DPP-4 inhibitor (n, %) | 11 (34.4) | 10 (31.3) | 1.000 |
|  | Meglitinide (n, %) | 1 (3.1) | 0 (0.0) | - |
|  | Thiazolidinedione (n, %) | 1 (3.1) | 3 (9.4) | 0.617 |
|  | SGLT-2 inhibitor (n, %) | 0 (0.0) | 0 (0.0) | - |
|  | Alpha glucosidase inhibitor (n, %) | 0 (0.0) | 1 (3.1) | - |
|  | Rapid acting insulin (n, %) | 1 (3.1) | 3 (9.4) | 0.480 |
|  | Basal insulin (n, %) | 7 (21.9) | 11 (34.4) | 0.289 |
|  | Pre-mixed insulin (n, %) | 7 (21.9) | 6 (18.8) | 1.000 |
|  | GLP-1 agonist (n, %) | 0 (0.0) | 0 (0.0) | - |
|  | Angiotensin Receptor Blocker (n, %) | 15 (46.9) | 14 (43.8) | 1.000 |
|  | Angiotension Converting Enzyme inhibitor (n, %) | 1 (3.1) | 5 (15.6) | 0.221 |
|  | Calcium channel blocker (n, %) | 11 (34.4) | 10 (31.3) | 1.000 |
|  | Diuretics (n, %) | 1 (3.1) | 4 (12.5) | 0.371 |
|  | Beta blocker (n, %) | 2 (6.3) | 2 (6.3) | 1.000 |
|  | Statin (n, %) | 12 (37.5) | 15 (46.9) | 0.628 |
|  | Fibrate (n, %) | 1 (3.1) | 0 (0.0) | - |
|  | Aspirin (n, %) | 6 (18.8) | 8 (25.0) | 0.724 |
|  | Clopidogrel (n, %) | 5 (15.6) | 5 (15.6) | 1.000 |
|  | Cilostazol (n, %) | 17 (53.1) | 11 (34.4) | 0.211 |

expressed as mean±SD, or n (%). by Paired sample t-test, or McNemar test.

DM indicates diabetes mellitus; BMI, body mass index; LDL, Low density lipoprotein; HDL, high density lipoprotein; BUN, blood urea nitrogen; AST, aspartate aminotransferase; ALT, alanine aminotransferase; GGT, gamma-glutamyl transferase; ALP, alkaline phosphatase; CAG, coronary angiography; DPP, dipeptidylpeptidase; SGLT, sodium-glucose transporter.

**Table S2.** Significantly differentiating metabolites between experimental groups including non-diabetic control, no DR, and DR subjects analyzed by GC-TOF-MS.

| **No.** | **t_R_(min)** | **Unique MS** | **Metabolite ^a^** | **AUC** | | **Fold Changes** | | |
| --- | --- | --- | --- | --- | --- | --- | --- | --- |
|  |  |  |  | **CON/no DR** | **DR/no DR** | **No DR/CON** | **DR/no DR** | |
| **Amino acids** | |  |  |  |  |  |  | |
| 1 | 7.47 | 174 | Glycine | 0.796 | 0.628 | 1.38* | 1.12 | |
| 2 | 9.33 | 232 | Aspartic acid | 0.948 | 0.561 | 0.46* | 0.91 | |
| 3 | 9.33 | 176 | Methionine | 0.853 | 0.510 | 1.81* | 0.98 | |
| 4 | 9.39 | 156 | Pyroglutamic acid | 0.842 | 0.510 | 0.64* | 0.93 | |
| 5 | 10.11 | 246 | Glutamic acid | 0.979 | 0.656 | 0.25* | 0.72* | |
| 6 | 10.54 | 116 | Asparagine | 0.931 | 0.588 | 2.30* | 1.09 | |
| 7 | 11.28 | 156 | Glutamine | 0.960 | 0.656 | 2.83* | 1.19* | |
| **Organic compounds** | | |  |  |  |  |  | |
| 8 | 5.05 | 117 | Lactic acid | 0.940 | 0.521 | 0.68* | 0.97 | |
| 9 | 6.94 | 189 | Urea | 0.699 | 0.648 | 1.22* | 1.13 | |
| 10 | 7.22 | 299 | Phosphoric acid | 0.770 | 0.590 | 1.11* | 1.05 | |
| 11 | 7.50 | 247 | Succinic acid | 0.908 | 0.527 | 0.60* | 1.02 | |
| 12 | 11.63 | 273 | Citric acid | 0.772 | 0.545 | 1.52* | 1.11 | |
| 13 | 13.51 | 441 | Uric acid | 0.716 | 0.530 | 1.43* | 1.03 | |
| **Carbohydrates** | |  |  |  |  |  |  | |
| 14 | 11.87 | 217 | 1,5-Anhydroglucitol | 0.939 | 0.553 | 0.25* | 1.20 | |
| 15 | 12.05 | 103 | Fructose | 0.858 | 0.519 | 2.02* | 1.03 | |
| 16 | 12.38 | 205 | Glucose | 0.921 | 0.540 | 1.37* | 0.97 | |
| 17 | 12.60 | 217 | Saccharide (1) | 0.928 | 0.600 | 2.70* | 1.10 | |
| 18 | 12.73 | 204 | Saccharide (2) | 0.920 | 0.630 | 1.85* | 0.92 | |
| 19 | 13.06 | 204 | Saccharide (3) | 0.838 | 0.536 | 2.28* | 0.89 | |
| 20 | 13.48 | 217 | myo-Inositol | 0.896 | 0.542 | 2.02* | 1.07 | |
| Metabolites were selected according to VIP >0.7 from OPLS-DA model | | | | | | | |  |
| ^a^ Tentatively identified metabolites by using HMDB, NIST, and in-house library | | | | | | | |  |
| * *p*-value<0.05 | | | | | | | |  |

**Table S3.** Significantly differentiating metabolites between experimental groups including non-diabetic control, no DR, and DR subjects analyzed by UPLC-Q-TOF-MS.

| **No.** | **Metabolites ^a^** | **t_R_ (min)** | **Measured MS (*m/z*)** | **HMDB formular** | **PPM** | **Adduct** | **AUC** | | **Fold Changes** | |
| --- | --- | --- | --- | --- | --- | --- | --- | --- | --- | --- |
|  |  |  |  |  |  |  | **CON/no DR** | **DR/no DR** | **No DR/CON** | **DR/no DR** |
| 21 | LysoPC 14:0 (sn-2) | 7.46 | 452.2776 | C_22_H_46_NO_7_P | 1.5 | [M-CH_3_]^-^ | 0.683 | 0.512 | 0.74* | 1.00 |
| 22 | LysoPC 20:5 (sn-2) | 7.57 | 526.2938 | C_28_H_48_NO_7_P | 1.0 | [M-CH_3_]^-^ | 0.740 | 0.577 | 1.58* | 0.85 |
| 23 | LysoPC 14:0 (sn-1) | 7.64 | 452.2782 | C_22_H_46_NO_7_P | 0.7 | [M-CH_3_]^-^ | 0.732 | 0.558 | 0.72* | 1.08 |
| 24 | LysoPC 20:5 (sn-1) | 7.71 | 526.2932 | C_28_H_48_NO_7_P | -1.3 | [M-CH_3_]^-^ | 0.718 | 0.565 | 1.49* | 0.88 |
| 25 | LysoPC 16:1 (sn-1) | 7.91 | 478.2932 | C_24_H_48_NO_7_P | 0.2 | [M-CH_3_]^-^ | 0.703 | 0.518 | 0.78* | 0.99 |
| 26 | LysoPC 22:5 (sn-2) | 8.24 | 554.3138 | C_30_H_52_NO_7_P | 2.0 | [M-CH_3_]^-^ | 0.823 | 0.601 | 1.34* | 0.90 |
| 27 | LysoPC 22:5 (sn-1) | 8.39 | 554.3246 | C_30_H_52_NO_7_P | 0.5 | [M-CH_3_]^-^ | 0.819 | 0.557 | 1.63* | 0.93 |
| 28 | LysoPC 16:0 (sn-2) | 8.47 | 480.3072 | C_24_H_50_NO_7_P | -2.1 | [M-CH_3_]^-^ | 0.913 | 0.504 | 0.79* | 0.99 |
| 29 | LysoPC 20:3 (sn-1) | 8.58 | 530.3224 | C_28_H_52_NO_7_P | 0.6 | [M-CH_3_]^-^ | 0.659 | 0.549 | 1.20* | 0.98 |
| 30 | LysoPC 18:1 (sn-2) | 8.72 | 506.3249 | C_26_H_52_NO_7_P | -0.6 | [M-CH_3_]^-^ | 0.797 | 0.515 | 1.22* | 1.01 |
| 31 | LysoPC 18:0 (sn-2) | 9.53 | 508.3398 | C_26_H_54_NO_7_P | 0.6 | [M-CH_3_]^-^ | 0.931 | 0.507 | 0.66* | 1.00 |
| Metabolites were selected according to VIP >0.7 from OPLS-DA model | | | | | | | | | | |
| LysoPC, lysophosphatidylcholine; two different forms of lysoPC were identified with the fatty acyl chain at sn-1 and sn-2 on the backbone of glycerol | | | | | | | | | | |
| ^a^ Tentatively identified metabolites by using HMDB, NIST, high resolution (PPM), and in-house library | | | | | | | | | | |
| * *p*-value < 0.05 | | | | | | | | | | |
